# Nuclear envelope and genome interactions in cell fate

**DOI:** 10.3389/fgene.2015.00095

**Published:** 2015-03-19

**Authors:** Jessica A. Talamas, Maya Capelson

**Affiliations:** Program in Epigenetics, Department of Cell and Developmental Biology, Smilow Center for Translational Research, Perelman School of Medicine, University of PennsylvaniaPhiladelphia, PA, USA

**Keywords:** nuclear envelope, nuclear pore, nuclear lamina, genome, cell fate, differentiation, gene regulation, nuclear organization

## Abstract

The eukaryotic cell nucleus houses an organism’s genome and is the location within the cell where all signaling induced and development-driven gene expression programs are ultimately specified. The genome is enclosed and separated from the cytoplasm by the nuclear envelope (NE), a double-lipid membrane bilayer, which contains a large variety of trans-membrane and associated protein complexes. In recent years, research regarding multiple aspects of the cell nucleus points to a highly dynamic and coordinated concert of efforts between chromatin and the NE in regulation of gene expression. Details of how this concert is orchestrated and how it directs cell differentiation and disease are coming to light at a rapid pace. Here we review existing and emerging concepts of how interactions between the genome and the NE may contribute to tissue specific gene expression programs to determine cell fate.

## Introduction

**FIGURE 1:**
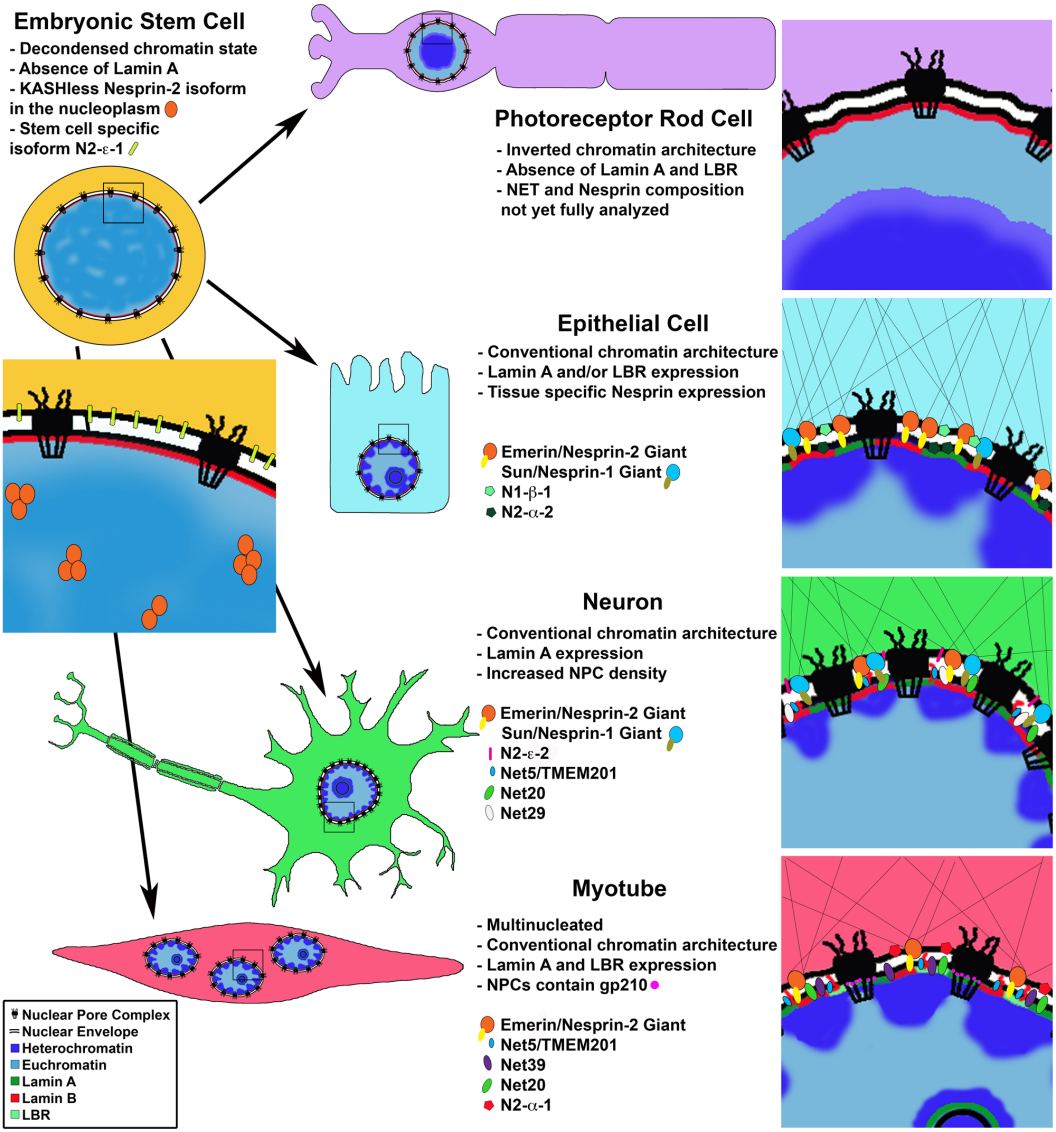
**Cell type specific changes in NE composition**. Cell differentiation coincides with changes in chromatin organization and protein composition of the nuclear lamina, nuclear envelope and nuclear pore complex. Photoreceptor rod cells lose expression of LBR and Lamin A leading to an inverted chromatin state with heterochromatin in the nuclear interior. Proteomic analysis of differentiated cells shows cell type specific nuclear envelope composition resulting in unique nucleo-cytoplasmic connections influencing cell morphology; and chromatin-NE interactions facilitating intra-nuclear genome reorganization and regulation of gene expression programs. Differentiated muscle cells uniquely express gp210 at the NPC leading to activation of muscle specific genes.

Pluripotent embryonic stem cells (ESCs) from many organisms display strikingly different chromatin structure and overall nuclear architecture when compared with differentiated cells (**Figure 1**). Microscopic visualization of DNA stains in ESC nuclei show diffuse staining indicative of a generally open chromatin state (Efroni et al., 2008; Ahmed et al., 2010). Consistent with this observation, comparisons of pluripotent stem cells with differentiated cells revealed changes in both levels and localization of epigenetic marks within the nuclear space (Bartova et al., 2008; Wen et al., 2009). Such cytological observations of the unique chromatin state of ESCs have been extensively confirmed by genome wide and functional studies of histone modifications and chromatin complexes (Mattout and Meshorer, 2010). Consistent with a decondensed and permissive chromatin state, pluripotent and totipotent cells exhibit higher chromatin mobility (Meshorer et al., 2006; Boskovic et al., 2014).

This large-scale change in overall chromatin structure, condensation and mobility during differentiation is supported by changes in nuclear structure and composition. During cell differentiation, individual genes, as well as larger chromosome regions are repositioned within the nuclear space, and this repositioning correlates with tissue specific gene expression profiles (Schneider and Grosschedl, 2007; Bickmore and van Steensel, 2013). Large-scale chromatin reorganization and gene repositioning during differentiation relies, at least in part, on losing or gaining interactions with major nuclear compartments such as the NE. Components of the NE, including the nuclear lamina, the nuclear membrane (NM) and the nuclear pore complex (NPC), come in close contact with the underlying genome and have been implicated in a number of chromatin-associated processes (Akhtar and Gasser, 2007; Arib and Akhtar, 2011; Van de Vosse et al., 2011; Amendola and van Steensel, 2014). While several of these processes have been characterized individually, how nuclear components work together to execute tissue specific gene expression programs is still unclear. In this review we aim to outline current understanding of the roles of major NE components in determining tissue specific cell fate and discuss selected examples illustrating their connection to genome organization and function.

## The Nuclear Lamina

The nuclear lamina is a meshwork of class V intermediate filament proteins lining the inner nuclear membrane (INM) of the NE (Prokocimer et al., 2009). The lamina is comprised of A and B type Lamins; Lamin A and C are the two major splice variants of a single gene (*LMNA*), while Lamin B1 and B2 are transcribed from distinct genes (*LMNB2* and *LMNB2*; Ho and Lammerding, 2012). Pre-Lamin A undergoes enzymatic cleavage to become mature Lamin A; and all Lamins are subject to a variety of post-translational modifications (Snider and Omary, 2014). Together with Lamin associated proteins, the Lamin filaments are known to provide structural support to the nucleus and to serve as a scaffold for spatial genome organization (Dittmer and Misteli, 2011). Specifically, Lamin proteins are known to function in tethering of heterochromatic and developmentally silenced domains to the nuclear periphery (Guelen et al., 2008; Ikegami et al., 2010; Peric-Hupkes and van Steensel, 2010; Peric-Hupkes et al., 2010), as well as interact with a myriad of proteins affecting chromatin organization and dynamics, such as transcription factors and chromatin remodelers (Ho and Lammerding, 2012). Notably, Lamins, particularly Lamin A, have also been visualized in the nuclear interior, often associated with nucleoli, another nuclear sub-compartment enriched in heterochromatin (Broers et al., 2005; Kind et al., 2013; Kind and van Steensel, 2014; Legartova et al., 2014; Padeken and Heun, 2014). While many mechanistic details remain unknown, it is becoming increasingly clear that Lamins play a pivotal role in the dynamic changes in chromatin and cellular organization required for determination and manifestation of cell fate.

### Temporal and Cell Type Specific Expression of Lamins

The B type Lamins (B1 and B2) are expressed in all cell types, while expression of Lamins A and C varies with cell type and developmental stage (Worman et al., 1988a; Rober et al., 1989). Immunofluorescence staining and immunoblotting with isotype specific anti-Lamin antibodies in mouse embryos show low expression of Lamin A/C in ESCs, which increases as cells differentiate (Constantinescu et al., 2006; Eckersley-Maslin et al., 2013). In mice, the increase in Lamin A/C expression is initiated as early as embryonic day 9 and as late as in the adult animal depending on the tissue type (Stewart and Burke, 1987; Rober et al., 1989). In direct support of a role for Lamin A in cell differentiation, experiments in mouse cells testing the effect of Lamin A levels on somatic to iPS cell reprogramming show that depletion of Lamin A accelerates the transition to pluripotency, while cells overexpressing Lamin A take longer to reprogram (Zuo et al., 2012).

Further supporting separate roles for A and B type Lamins, studies of Lamin filaments in amphibian oocytes and HeLa cells indicate that Lamins A, B and C form discrete, but interconnected, lattice structures with differing physical properties (Goldberg et al., 2008; Shimi et al., 2008; Kolb et al., 2011). In agreement with these studies, immunofluorescence staining in mouse embryonic fibroblasts (MEFs) shows non-uniform staining of the nuclear envelope/lamina where Lamin B and Lamin A do not overlap (Legartova et al., 2014). Direct evidence for tissue specific function of Lamin proteins comes from mutations in the human *Lamin A* (*LMNA*) gene, which lead to an array of serious diseases called laminopathies, including cardiomyopathy, muscular dystrophy, lipodystropy, neuropathy and progeria (Dittmer and Misteli, 2011). Together, these data demonstrate that Lamins are expressed in a tissue specific manner and form unique territories in the lamina likely contributing to cell type specific NE composition (**Figure 1**), and support the notion that Lamins play functional roles in cell differentiation, as discussed further below.

### Lamins Maintain Heterochromatin at the Nuclear Periphery

Microscopic observations of somatic cell nuclei indicate that in most cell types, heterochromatin is enriched at the nuclear periphery and this enrichment becomes more pronounced with cell differentiation (Wu et al., 2005; Reik, 2007; Ueda et al., 2014). Known epigenetic marks of heterochromatin commonly found at the nuclear periphery include H3K9me1, H3K9me2, H3K9me3, H3K56me3, H4K20me2, H4K20me3, H3K27me2, H3K27me3, and H3K4ac (Eberhart et al., 2013). Reported genome wide Chromatin Immunoprecipitation (ChIP) of the heterochromatin mark H3K9me2 shows that coverage of “large organized chromatin K domains” (LOCKS) grows from 17.5–24% in pluripotent human stem cells to 39.3–44.8% in differentiated cell lines (Wen et al., 2009, 2012). This data combined with DNA adenine methyltransferase identification (DamID) studies of Lamin B1 Associated Chromatin Domains (LADS), exhibits a significant overlap between LOCKS and LADs, which supports a role for Lamin B1 in the peripheral localization of these heterochromatic domains (Guelen et al., 2008; Peric-Hupkes et al., 2010; Amendola and van Steensel, 2014), and agrees with the visually observed changes in chromatin organization during differentiation.

An especially impressive example of the requirement for Lamin expression in heterochromatin organization during cell differentiation comes from studies of retinal rod cells in nocturnal mammals. The authors noticed the conventional nuclear architecture described for most cell types, with heterochromatin lining the nuclear periphery and euchromatin in the nuclear interior, is essentially reversed in retinal photoreceptor rod cells (Solovei et al., 2009). This inverted architecture is thought to have evolved to channel light more efficiently in the eye and has provided a unique and fruitful system, in which to study basic requirements for spatial organization of chromatin.

In a series of elegant experiments the authors demonstrate that during cell differentiation, conventional chromatin architecture requires the sequential expression of first the NE transmembrane protein Lamin B receptor (LBR) and then its replacement by Lamin A/C, with some cell types expressing both proteins (Solovei et al., 2013). The chromatin architecture inversion, with euchromatin at the nuclear periphery and heterochromatin in the nuclear center, in photoreceptor nuclei is a result of loss of expression of both Lamin A/C and LBR from the nuclear envelope (**Figure 1**). They further show this loss and the subsequent chromatin rearrangements coincide with terminal differentiation of the rod cells. Strikingly, the inversion phenotype was successfully recapitulated experimentally in additional cell types, such as the hair follicle (which does not express Lamin A/C), using LBR null mice, and examination of double null (*Lbr-/- Lmna-/-*) mouse pups indeed showed an inverted phenotype in all post-mitotic cell types studied. Conversely, artificially maintaining expression of LBR, but not Lamin C in these cells was enough to prevent chromatin inversion, suggesting that Lamin C does not bind chromatin directly but perhaps via other nuclear envelope associated proteins such as LEM domain proteins (discussed further below). Although it is not presently clear how the conventional versus the inverted heterochromatin architecture affects cell type specific gene expression, these results support the notion that Lamins B and A/C are needed to position heterochromatin in a cell type specific manner.

### Lamins Recruit Differentiation-Specific Genes

**FIGURE 2:**
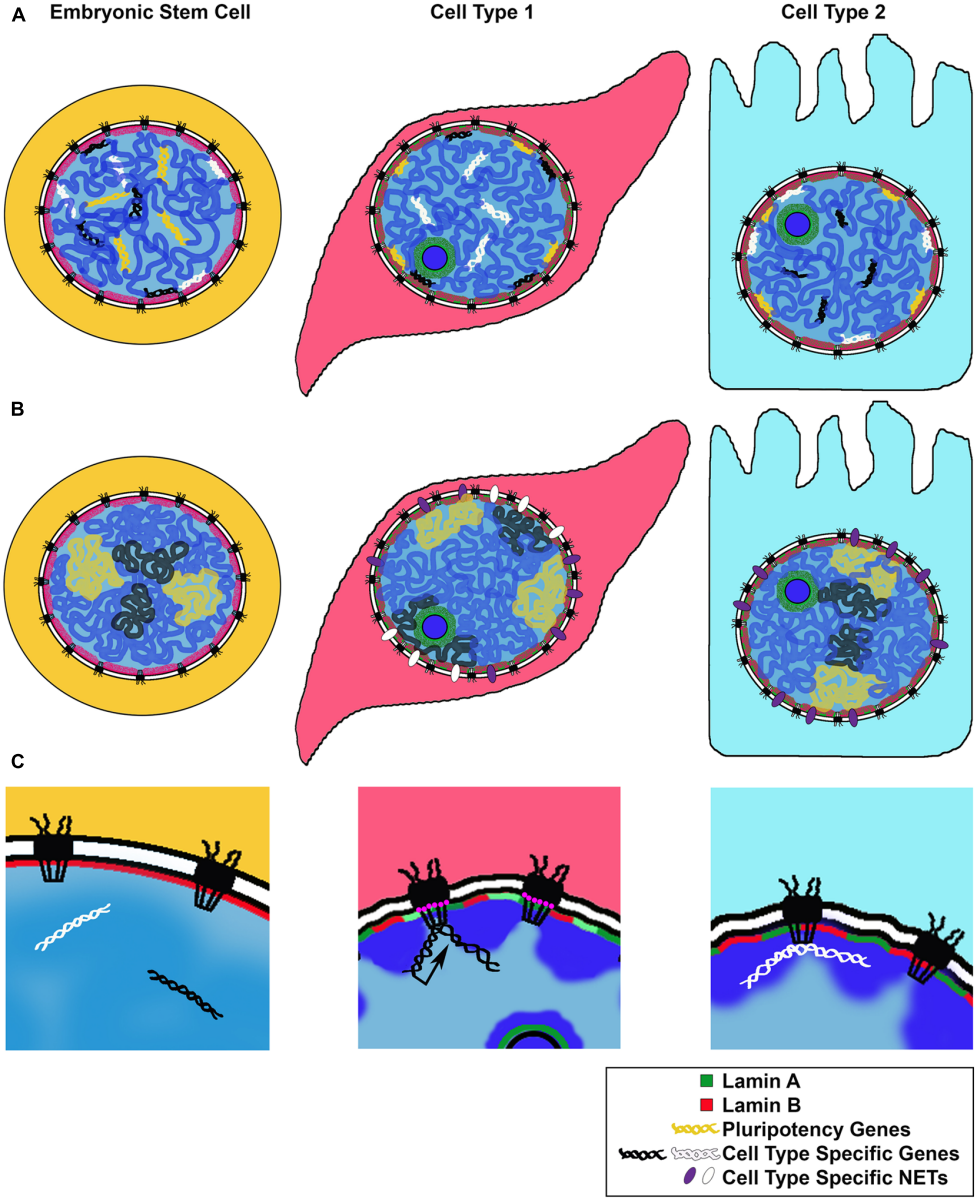
**Models for changes in NE-genome interactions during differentiation**. We illustrate three proposed mechanisms for genomic rearrangement during cell differentiation. **(A)** Repositioning of cell type specific genes: along the steps to a fully differentiated cell, genes required for pluripotency or an alternate differentiation pathway are repositioned to the transcriptionally repressive nuclear periphery. Genes required for differentiation or cell type maintenance are kept in the nuclear interior. **(B)** Expression of cell type specific NETs during cell differentiation repositions chromosomes or nuclear territories to the nuclear periphery, influencing their transcriptional activity. **(C)** Cell type specific genes can be repositioned to the NPC for transcriptional activation (black arrow) or other regulation (white), such as establishment of chromatin boundaries or non-expressed genes; NPC composition may change depending on the cell type, with some Nups, such as Nup210 (pink circles), expressed only in certain differentiated states.

Genome wide studies of LADs during neuronal differentiation in mice showed that while ESCs and terminally differentiated cells share a broad LAD structure, smaller sub regions of gene clusters undergo rearrangements corresponding to steps of the differentiation process (Amendola and van Steensel, 2014; Luperchio et al., 2014). For example, genes associated with “stemness,” as well as cell cycle related genes, become lamina-associated during differentiation. Conversely, cell type or lineage specific genes were released from the lamina and de-repressed or “unlocked” for expression at a subsequent step in differentiation (Peric-Hupkes et al., 2010; **Figure 2A**).

In support of a role for Lamins in differentiation-specific gene expression programs, B type Lamin knockout mouse models display an array of organogenesis defects, particularly in the brain, yet self-renewal and pluripotency properties of mouse ESCs are not affected (Kim et al., 2011). In *Drosophila*, the gene encoding a critical transcriptional factor *hunchback* was shown to move to the nuclear lamina during differentiation of neuroblast cells to neurons (Kohwi et al., 2013). This gene repositioning correlated with a loss of progenitor cell competence and was found to be dependent on the B type Lamin Dm0. Depletion of Lamin Dm0 extended neuroblast competence, presumably through disruption of targeting the *hunchback* locus to the nuclear lamina. These studies indicate the nuclear lamina is extensively utilized throughout metazoa to stably silence differentiation-specific genes.

How do Lamins bind to heterochromatin or developmentally silenced genes? In addition to reports of a DNA binding domain in Lamin A (Bruston et al., 2010) and *in vitro* interactions of Lamins with DNA and histones (Taniura et al., 1995; Stierle et al., 2003), there are several examples of Lamins interacting with chromatin binding NE proteins, chromatin regulatory machinery and transcriptional regulators. For example, interactions between the lamina and constitutive highly condensed heterochromatin are thought to be mediated via LBR and heterochromatic proteins such as Heterochromatin Protein 1 (HP1), discussed in further detail above and below. Additionally, a recent study identified a new mediator of Lamin-genome interactions, which appears to be utilized by silenced genes in mouse fibroblasts (Zullo et al., 2012). The authors have characterized discrete DNA sequences within LADs spanning the IgH and Cup3a genes able to position these loci to the nuclear lamina and concomitantly silence gene activity. These recurring lamina-associated sequences (LASs) were found to be enriched for a GAGA motif and to bind the transcriptional repressor cKrox in a complex with histone deacetylase 3 (HDAC3) and the lamina-associated NE protein Lap2β. The cKrox/HDAC3/Lap2β complex is necessary for tethering of LAS-containing target genes to the lamina, and represents another key molecular explanation for the coupling of nuclear localization and transcriptional repression. These findings are consistent with a previous study demonstrating the ability of Lap2β to reposition an ectopic binding site to the nuclear periphery and silence expression of genes near the binding site (Finlan et al., 2008). In this example, Lap2β was fused to the bacterial LacI protein, which binds the *lactose operon* (*lacO)* repeats array, introduced into the genome of human culture cells, and the ability of the Lap2β-LacI to silence genes near its target site was similarly found to be dependent on HDAC activity.

On a cautionary note, initial DamID studies of Lamin-chromatin binding sites required a population of cells, and thus the resulting LADs are reflections of both an average of many cells in a population as well as an amalgamation of binding events acquired over the time a DamID fusion protein is expressed. When these studies were repeated using the m6A-tracer technique which is able to label stochastic protein-Dam chromatin interactions in single, living cells, the authors found that at a given time only a subset of the initially described LADs was localized to the periphery while the rest were often located in the nuclear interior and further, this subset often changed following each cell division (Kind et al., 2013; Kind and van Steensel, 2014). Use of the m6A-tracer technique to specifically monitor Lamin A-chromatin binding shows Lamin A binding at the nuclear periphery and also around the nucleoli. These results support previous observations of an intranuclear pool of Lamin A (Moir et al., 2000a,b) and indicate a stochastic nature of Lamin-chromatin binding, which would allow for dynamic binding of LAD sequences to either A or B type Lamins, or Lamin associated proteins, as needed.

## The Nuclear Membrane

The nuclear envelope is a double lipid bilayer system made of the INM, directly adjacent and connected to the Lamin filaments, and the outer nuclear membrane (ONM), which is contiguous with the endoplasmic reticulum. The space between these membranes is called the perinuclear space (PNS) and is interrupted by NPCs which fenestrate the NM. Originally viewed as simply a protective barrier for the genome, the nuclear envelope along with its nuclear envelope transmembrane proteins (NETs) and associated soluble proteins are now known to participate in an array of cellular functions including genome organization, nuclear migration and positioning, cell cycle regulation, signaling, and cell differentiation (Dauer and Worman, 2009; Chow et al., 2012; Gomez-Cavazos and Hetzer, 2012). While the NM is now accepted as a dynamic interface between the nucleus and cytoplasm, exactly how the NM and its composite proteins are manifesting these processes is still largely unclear. An exciting current area of nuclear study is analysis of the nuclear envelope proteome and characterizing functions of NE proteins in more detail.

### The Nuclear Membrane Proteome is Tissue Specific

To date the NE/NM proteome has been analyzed in three tissues – liver (Schirmer et al., 2003; Korfali et al., 2012), muscle (Wilkie et al., 2011) and blood leukocytes (Korfali et al., 2010), as well as mouse neuroblastoma cells in culture (Dreger et al., 2001). The three most recent of these studies were performed under identical experimental conditions and therefore the resulting data sets can be directly compared. These studies identified 1,037 NETs in total, a huge increase compared with only 67 potential NETs known in 2003. The results indicate a surprisingly high degree of tissue specificity in NE protein composition with only 16% of identified transmembrane proteins shared between the three tissues (**Figure 1**). These tissue specific results were directly verified for several novel NETs by immunofluorescence staining and comparison with known tissue specific expression profiles (Korfali et al., 2010, 2012; Wilkie et al., 2011). Further highlighting cell type specific expression of these proteins, in tissues composed of multiple cell types, often, only a subset of cells displayed a clear nuclear rim staining for a given NET (Korfali et al., 2012). Additionally, results of these proteomic analyses correlate with previously annotated protein complexes, reported in the interaction networks by the Johns Hopkins Human Protein Reference Data (HPRD) database. The authors found a preference for NETs proposed to act in a complex according to the interactome data, to have similar tissue type expression profiles (Korfali et al., 2012).

### Nuclear Membrane Proteins Reposition Chromosomes

Early work on NETs focused in large part on their role in NE reassembly following cell division. One important outcome of these studies is the finding that many NETs are able to directly bind mitotic chromatin (Ulbert et al., 2006). This finding becomes relevant in the context of cell fate determination as it indicates these NETs have the capacity to bind chromatin also in interphase and thus are able to contribute to three dimensional genome organization and gene expression programs. In addition to the LBR and the INM protein Lap2β examples provided above, other NETs have been found to directly reposition genomic loci to the NE/nuclear lamina. For example, a domain of the NET Emerin, fused to LacI, repositioned a *lacO* array to the INM, and interestingly, this repositioning was found to require passage through mitosis (Reddy and Singh, 2008; Reddy et al., 2008). Similarly, NE targeting by the LacI-Lamin B fusion was found to require cell division (Kumaran and Spector, 2008; Kumaran et al., 2008), suggesting that cells have to break down their nuclear architecture to allow reorganization of NE-genome contacts. Observed redistribution of LAD subsets between the nuclear interior and periphery after mitosis lends further support to this idea (Kind et al., 2013). In terms of cell fate specification, these results suggest that cell cycle exit could effectively “fix”/make static one’s nuclear genome organization.

A visual screen for the effects of NETs on chromosome positioning was performed for 22 novel NETs identified from the liver specific proteomic analysis (Korfali et al., 2012) as well as the more familiar NET, Emerin (Zuleger et al., 2013). The ability of transiently expressed NETs to reposition chromosomes was assayed, using chromosome paint and image analysis, in human cell culture. Four of the tested proteins, NET5, NET29, NET39, and NET47 were able to specifically reposition both copies of chromosome 5 to the nuclear periphery. Only NET29 and NET39 had an effect on chromosome 13, and none of the NETs tested effected nuclear positioning of either chromosome 17 or 19. In support of tissue specific chromosome positioning via tissue specific NET expression, the authors correlate peripheral localization of chromosome 5 in liver tissue with preferential expression of NET47 (70% of total expression across tissue types). In kidney cells, which account for only 3% of NET47 total expression, chromosome 5 is found more often in the nuclear interior. Importantly, the authors show by RNAi knockdown that NET positioning of chromosomes at the nuclear periphery is reversible.

This study yields several important conceptual findings: firstly, it provides examples of tissue specific NET expression, giving rise to unique NE compositions correlating with cell type (**Figure 1**). Secondly, these results suggest that NETs bind specific chromosomes in a reversible manner, linking chromosome positioning with differentiation (**Figure 2B**). In this manner, tissue specific NETs may function to reposition entire chromosomes or large chromosomal regions to the nuclear periphery, which may further assist or stabilize the silencing of specific developmental genes by association with the nuclear lamina (**Figure 2A**). Thirdly, multiple NETs can act to position the same chromosome, perhaps via cellular regulation of relative abundance of different NETs. Lastly, in addition to tissue specific expression levels, several of the NETs in this study appear to have tissue specific splice variants. Together these provide another layer of regulation to how a cell might fine-tune its gene expression profile during differentiation by utilizing NETs to position chromosomes at the NE in a tissue specificmanner.

### Nuclear Membrane Proteins Regulate Chromatin State

Lamin B receptor, discussed above, is an INM protein shown to interact directly with Lamin B and the chromodomain heterochromatic protein HP1 (Worman et al., 1988b; Schuler et al., 1994; Ye and Worman, 1996; Ye et al., 1997). Initial characterization of LBR indicates it forms oligomeric structures which, in contrast to the smooth nuclear rim staining observed for Lamin proteins, localize into discrete microdomains in the NE (Makatsori et al., 2004). More recent experiments using a Celluspots peptide array of 384 histone tail peptides showed the nucleoplasmic domain of LBR binds a specific set of heterochromatin marks, namely H4K20me2, H4K20ac, H4R19me2s, H4R19me2a and H4R23me2s (“a” and “s” refer to arginine methylation patterns: asymmetric or symmetric, respectively; Hirano et al., 2012). To verify these binding partners *in vivo* the authors showed the ChIP fraction obtained using an anti-LBR antibody was significantly enriched for H4K20me2 and that this heterochromatin mark indeed localized to the nuclear periphery. As H4K20me2 is widespread throughout the genome, binding of LBR to the additional, less common, methylated histone residues provides a possibility for further specificity in tethering unique heterochromatin or developmentally silenced sites to the nuclear periphery. Fluorescence recovery after photobleaching (FRAP) experiments analyzing mobility of LBR truncation mutants revealed domains involved in interactions with histone H4, but not with Lamin B1 or B2 are required for formation of stable LBR microdomains in the NE. To investigate a role for LBR in heterochromatin formation, *in vitro* experiments using atomic force microscopy to measure chromatin compaction showed incubation of recombinant LBR with reconstituted chromatin resulted in highly aggregated chromatin fibers compared with controls. This study further demonstrated that LBR itself has the ability to repress transcription of a reporter plasmid.

Together with the previously discussed role for LBR in maintaining a conventional chromatin architecture (Solovei et al., 2013) and reports of *in vivo* effects of LBR depletion or mutation (Worman, 2005), the study described above suggests a differentiation specific function for LBR in formation and maintenance of heterochromatin at the nuclear periphery via roles in chromatin compaction and transcriptional repression. LBR provides a clear example of a NET physically and functionally bridging Lamins to heterochromatin at the nuclear face ofthe INM.

### Nuclear Membrane Proteins are Linked to the Cytoskeleton

Thus far we have discussed changes within the nucleus that lead to or occur with changes required for cell fate determination. However, often during cell differentiation there are significant physical changes in cell shape and size as well as in nuclear positioning, and sometimes the formation of multinucleate cells. Almost a decade ago a complex physically linking the nucleoskeleton and cytoskeleton (the LINC complex) was first described (Padmakumar et al., 2005; Crisp et al., 2006). The finding that Lamin binding proteins of the INM interact with cytoskeletal binding proteins of the ONM via the PNS was the first evidence of NPC independent communication between the nucleus and cytoplasm. The SUN proteins are INM specific with their SUN domain extending into the PNS. The SUN domain interacts with the C-terminal KASH (Klarsicht-ANC-Syne-homology) domain of Nesprins, extending in most cases, from the ONM into the PNS. Nesprins are further structurally characterized by a spectrin repeat rod domain and a variable N-terminal domain which interacts with cytoskeletal elements including actin and plectin (Wilhelmsen et al., 2005). To date the LINC complexes have been implicated in a variety of cell processes including nuclear size, shape and positioning, cell migration and polarity as well as mechano-sensory signal transduction (reviewed in Lombardi and Lammerding, 2011; Razafsky et al., 2011; Neumann and Noegel, 2014) In addition to roles in NE embedded LINC complexes at the ONM, Nesprin-2a, lacking a transmembrane domain has been shown to exist within the nuclear interior and Nesprin-2 has been shown to directly interact with Lamin A (Haque et al., 2010;Yang et al., 2013).

The Nesprin protein family continues to grow with the four Nesprin coding genes currently described in mammals giving rise to an ever-increasing number of isoforms (Apel et al., 2000; Wilhelmsen et al., 2005; Zhang et al., 2005; Roux et al., 2009). Evolutionary conservation analysis of these gene sequences indicates they are the result of two whole-gene duplication events followed by individual rearrangements (Simpson and Roberts, 2008). Both Nesprin-1 and Nesprin-2 genes have internal promoters which give rise to shorter isoforms. At present while Nesprin-3 has two known isoforms, Nesprin-1 has 21 identified isoforms and Nesprin-2 has 14. While only one Nesprin-4 variant has been reported, its expression appears specific to secretory epithelial cells.

Of the known LINC complex components, expression of both SUN and Nesprin proteins appear to exhibit temporal and tissue specific expression patterns (**Figure 1**; Randles et al., 2010; Razafsky et al., 2013). A recent study of the expression patterns of Nesprin isoforms in a panel of 20 human tissues and 7 human cell lines (including ESCs) reveals complex expression profiles of Nesprin isoforms (Duong et al., 2014). Quantitative PCR was used to examine the distribution of expression of nine Nesprin isoforms from Nesprin-1 and Nesprin-2. The results indicate unique Nesprin profiles for each tissue or cell line. Perhaps expectedly, ESCs display a unique “Nesprinome” void of Nesprin-1 isoforms. ESCs predominantly express Nesprin-2 giant as well as the two smaller isoforms N2-e-1 and N2-a-2. Of further interest, when localization of Nesprin-2 giant was examined in ESCs, rather than the nuclear rim localization observed in differentiated cell types, the protein was visualized within the nucleoplasm. The authors found that this Nesprin-2 species lacks the KASH domain revealing a novel nucleoplasmic role for this protein. Nesprin isoform distribution in differentiated tissues was highly variable. For example, liver tissue was reported to have 95% relative abundance of Nesprin-2 giant, while heart tissue has 36% and brain only 8%. While more work is needed, these results suggest an important role for Nesprins in determining cell identity and the transition from Nesprin-1 to Nesprin-2 isoforms as a signature of cell differentiation.

A separate study of Lamin, SUN and Nesprin expression profiles in the developing mouse central nervous system confirmed many of the conclusions made above (Razafsky et al., 2013). The authors found unique expression profiles for all three of these protein families corresponding to differentiation stage and cell type. Notably they found that as differentiation progressed, lower molecular weight isoforms of the Nesprin giants became predominant. They additionally confirmed the presence of KASH-less isoforms of Nesprin1 in CNS tissues.

One can imagine a model where Nesprin isoforms are expressed in response to developmental signals and then themselves confer cytoskeletal changes as well as alter 3D organization of the genome to promote further tissue specific gene expression. They can do so via relaying these signals to INM proteins or through their own, yet undetermined, nucleoplasmic roles. Additionally, changes in nuclear size, shape and relative position within the cell can potentially influence the kinetics of nuclear processes and thus gene expression. Perhaps the most exciting implication of the LINC complex lies in its connection of the chromatin-associated INM proteins to cytoskeletal proteins via Nesprins, suggesting that cytoplasmic forces can directly move or alter nuclear chromatin positioning. This idea is supported by studies in both *Caenorhabditis elegans* and mice functionally linking cytoskeletal components to proper chromosome pairing and movement, as well as telomere clustering during meiosis via Sun/KASH protein bridges (Sato et al., 2009; Morimoto et al., 2012; Horn et al., 2013; Woglar and Jantsch, 2014). The large number of NE proteins and their splice variants, expressed in a tissue specific manner, connecting chromatin to the cytoskeleton, provide a window into the complex and interconnected mechanisms utilized by the cell nucleus to manifest its ultimate destiny (**Figure 1**).

## The Nuclear Pore Complex

The NPCs are multi-component protein complexes that form selectively permeable channels through the NE. The primary function of the NPCs is to mediate nucleo-cytoplasmic transport of molecules and thus allow communication between the nucleus and the cytoplasm (Wente and Rout, 2010; Raices and D’Angelo, 2012). They are estimated to be the largest protein complexes in the cell, ∼90–120 MDa in human cells. The NPC is composed of multiple copies of ∼30 individual components, termed Nucleoporins (Nups). The overall structure of the NPC is highly conserved and displays an eightfold rotational symmetry. Its core consists of a ring of membrane-embedded scaffold sub-complexes built around a central transport channel. The NPC core is further connected to its auxiliary structures, such as the meshwork of phenylalanine glycine repeat containing Nups (FG Nups), which fill the central channel and form the permeability transport barrier, the cytoplasmic filaments and the nuclear basket, which extends into the nuclear space (D’Angelo and Hetzer, 2008). Interestingly, individual Nups display highly variable rates of association with the nuclear pore (Rabut et al., 2004). While the core scaffold Nups have been shown to be remarkably stable once assembled into the NPC, with residence times exceeding one cell cycle, many of the non-scaffold Nups, such as the FG Nups and Nups of the nuclear basket were found to be highly dynamic, able to move on and off the pore with kinetics of seconds to a few hours.

Via its transport functions, the NPC plays an obvious role in gene regulation by controlling export of generated RNA and import of transcription and signaling factors. Yet, in addition to its canonical transport role, the NPC and individual Nups have been shown to play a role in genome organization and gene expression via direct binding to specific genomic locations (Casolari et al., 2004; Taddei et al., 2006; Brown et al., 2008; Capelson et al., 2010; Kalverda et al., 2010; Vaquerizas et al., 2010; Ikegami and Lieb, 2013; Liang et al., 2013; Ptak et al., 2014; Sood and Brickner, 2014). Multiple studies in a variety of genomes have identified the presence of specific Nups at active and silent genes, and have revealed a functional requirement for NPC components in execution or maintenance of select transcriptional programs and chromatin states, as detailed below. Additionally, a number of Nups have been demonstrated to be critical for certain paths of tissue specific differentiation. An intriguing possibility that arises from these studies is the potential ability of the NPC to integrate its transport and genome-binding roles, bridging for instance, the nuclear import of developmental transcription factors to their activating function at target promoters. In this manner, the NPC has emerged as a new scaffold for genome organization, and may play a role as a nexus of developmental signaling, able to coordinate transport, spatial genome organization and gene expression.

### Nuclear Pore Proteins Drive Tissue Specific Differentiation

Tissue specific expression of Nups has not been systematically analyzed in mutli-cellular organisms, but many individual examples that point to tissue specific roles of Nups have been reported. For instance, Nup50, a dynamic Nup, is highly expressed in the mammalian neural tube and the testis, particularly in the male germ cells (Trichet et al., 1999; Smitherman et al., 2000), while Nup45 exhibits variable expression in select mouse and rat cell lines (Hu and Gerace, 1998). Several Nups have been reported to change expression during cardiomyocyte differentiation (Perez-Terzic et al., 2003), as well as in response to cardiac hypertrophy (Chahine et al., 2015). Publically available genome wide expression studies in various cell types and organs also readily show differential expression of Nups. For instance, RNA Sequencing (RNA-Seq) and *in situ* RNA hybridization studies of the early *Drosophila* embryo revealed that Nups vary in their expression patterns relative to embryonic segments and developmental time points (Combs and Eisen, 2013), suggesting that different Nups are linked to different developmental pathways.

Strikingly, a number of tissue specific pathologies in humans and tissue specific phenotypes in model organisms have been described for mutations in a variety of both stable and dynamic Nups (Capelson and Hetzer, 2009; Xu and Powers, 2009; Raices and D’Angelo, 2012). For example, inherited cases of a cardiac disorder atrial fibrillation have been mapped to a missense mutation in the human Nup155, a stable Nup, which is highly expressed in the heart, liver and skeletal muscle (Zhang et al., 2008). Additionally, a mutation in the FG Nup Nup62 has been shown to underlie the familial form of infantile bilateral striatal necrosis (Basel-Vanagaite et al., 2006). Nup133, another stable Nup of the NPC scaffold, was found to be required for neuronal differentiation in the mouse embryo, and ESCs carrying a functionally null mutation in Nup133 are not able to undergo terminal differentiation into neurons (Lupu et al., 2008). Interestingly, a component of the same NPC scaffold sub-complex, ELYS, affects neuronal, retinal and intestinal development and proliferation in zebrafish (Davuluri et al., 2008; de Jong-Curtain et al., 2009). A large number of plant Nups, including Nup96, Nup160, ELYS and Tpr, have been reported to affect a diverse array of tissue specific processes, such as flowering, hormone signaling and immune function (Meier and Brkljacic, 2009). In *Drosophila*, several Nups, including Nup98/Nup96, Seh1 and Nup154 were uncovered to play a role in gametogenesis (Gigliotti et al., 1998; Parrott et al., 2011; Senger et al., 2011), where mutations in these Nups disrupt germ cell differentiation and cause sterility in males and females. In *C. elegans*, Nups such as the homolog of Nup98 were demonstrated to be critical for the formation of germline-specific P granules (Voronina and Seydoux, 2010), and multiple Nups have been shown to be required for normal embryonic development (Galy et al., 2003). Knowledge of the molecular mechanisms behind most of these developmental defects remains incomplete, and the connection of the NPC to chromatin organization may provide a new perspective to understanding these phenotypes.

Perhaps the most remarkable and well characterized example of a nuclear pore component playing a role in differentiation is that of Nup210. The transmembrane nucleoporin Nup210 is absent in mouse progenitor myoblasts and ESCs, but its expression is sharply upregulated during differentiation of these lineages into myotubes and neuroprogenitors, respectively (D’Angelo et al., 2012). Nup210 was further shown to be functionally necessary for these differentiation events, suggesting the NPC undergoes a compositional change required for the developmental programs of these cell types. Interestingly, the general transport properties of the NPC appear to remain unchanged by the addition of Nup210. Yet the expression of a subset of developmental genes was found to be dependent on Nup210 during myogenesis, indicating again a possible role of an NPC component in direct gene regulation to specify cell fate (**Figure 1**).

Wnt signaling, a central developmental signaling pathway of multi-cellular organisms, has also been repeatedly linked to the nuclear pore (Sharma et al., 2014). Wnt signaling relies on β-catenin as the primary transducer of activating signals from the plasma membrane to the nucleus, resulting in regulated shuttling of β-catenin between the nucleus and the cytoplasm. Nuclear import of β-catenin has been shown to be independent of the normal nuclear localization signal (NLS)/importins-regulated transport, and instead to involve direct interactions with a number of FG Nups, such as Nup62 and Nup358 (Sharma et al., 2012). Once in the nucleus, activated β-catenin associates with transcription factors of the LEF-1/TCF family and together, they induce transcription of Wnt target genes. One such member of the LEF-1/TCF family, TCF-4 has been shown to be sumoylated by Nup358, which carries a SUMO E3 ligase activity, and this sumoylation increases the transcription activity of TCF-4 and its binding to β-catenin (Shitashige et al., 2008). An additional key component of the Wnt pathway, APC, which is required for stabilizing and thus activating β-catenin, has been similarly reported to interact with specific FG Nups, such as Nup153 and Nup358 (Collin et al., 2008; Murawala et al., 2009).

These findings illustrate that Wnt pathway components are regulated by FG Nups both in terms of transport and function. Given the indispensable nature of Wnt signaling in stem cell maintenance, embryonic development and cell migration, these connections heavily implicate Nups in both normal development and oncogenic transformation. Intriguingly, the pluripotency state itself has been postulated to be regulated by the NPC via controlling levels of the pluripotency factors Oct4, Sox2 and Nanog in the nucleus (Yang et al., 2014). Together, these studies underscore the functional roles of the NPC in regulating developmental states and transitions. The mechanisms of these roles will be a fruitful subject for future investigations in the field’s efforts to fully understand cell fate determination.

### Nuclear Pore Proteins Facilitate Transcription

The phenotypes of Nups in tissue specific differentiation, described above, can result from either the transport or the genome regulatory roles of Nups, or possibly, from the integration of both. Multiple examples of cell type specific transport have been reported, and proposed transport mechanisms of Nups in development have been reviewed recently (Hogarth et al., 2005; Xylourgidis and Fornerod, 2009; Raices and D’Angelo, 2012). Here, we concentrate on recent work on the emerging roles of Nups in transcription and chromatin function, which may provide an alternative mechanism for the tissue specific roles of the NPC.

A functional relationship between nuclear pores and nuclear organization of chromatin was originally proposed based on EM close ups of mammalian nuclei that show frequent association of what appears to be decondensed chromatin with nuclear pores (Capelson and Hetzer, 2009). Such lighter stained, decondensed chromatin is thought to correspond to active regions of the genome that are more permissive to transcription. The observed correlation between NPCs and open/active chromatin was the basis for the ‘gene gating hypothesis’ (Blobel, 1985), which proposed that NPCs preferentially interact with and possibly regulate active genes to promote coregulation of transcription and mRNA export. Such images also suggested that the NPCs somehow participate in the establishment or maintenance of decondensed active chromatin.

A large amount of work in the yeast system has provided evidence for the role of the NPC in transcriptional activation. Genome wide studies in *Saccharomyces cerevisiae* demonstrated that some Nups, such as Mlp1, Nup2 and Nup60 often occupy regions of highly transcribed genes (Casolari et al., 2004, 2005), and revealed an interaction between the NPC component Nup2 and promoters of select active genes, termed the “Nup-PI” phenomenon (Schmid et al., 2006). Inducible yeast genes such as INO1, GAL and HXK1 are targeted to the NPC upon activation, and this association has been shown to be functionally important (Taddei et al., 2006; Light et al., 2010). Mechanistically, NPC-genome contacts in yeast have been shown to involve components of the histone acetyltransferase (HAT) SAGA complex (Rodriguez-Navarro et al., 2004; Cabal et al., 2006; Luthra et al., 2007), and mRNA export complexes TREX2 and THO-TREX (Rougemaille et al., 2008), as well as a transcription factor Put3 (Brickner et al., 2012).

A recently proposed function of the NPC-gene interactions that is especially relevant to cell fate control is a potential role in epigenetic memory of transcriptional events. The inducible yeast genes INO1, GAL and HXK1 have been shown to remain associated with the NPC for multiple generations, following their initial induction and during subsequent repression (Tan-Wong et al., 2009; Light et al., 2010). Interestingly, this association with the NPC was found to be important for the enhanced transcriptional response during reinduction, suggesting that binding of the NPC to recently transcribed genes primes them for later reactivation and in this manner, serves as a memory mark of transcriptional events. For GAL1 or HXK1, the maintenance of this transcriptional memory was found dependent on the nuclear basket Nup Mlp1, a homolog of the mammalian Nup Tpr (Tan-Wong et al., 2009). For INO1, it requires binding of Nup100 (mammalian Nup98), as well as changes in chromatin structure of the gene promoter, such as incorporation of the histone variant H2A.Z (Light et al., 2010).

In metazoa, the roles of the NPC in transcriptional activation, chromatin structure and epigenetic memory should be particularly important for tissue specific development. In support of this idea, several studies have analyzed genome wide chromatin binding of Nups in *Drosophila* and reported binding of a subset of fly Nups to developmental genes (Capelson et al., 2010; Kalverda and Fornerod, 2010; Vaquerizas et al., 2010). In the fly genome, Nups such as Nup98, Sec13, Nup50 and FG Nups such as Nup62 are recruited to loci actively transcribed by RNA Polymerase II (RNAP II) or to genes undergoing developmental induction, where they were found to be functionally necessary for full activation (Capelson et al., 2010; Kalverda and Fornerod, 2010). Additionally, Nups of the nuclear basket such as Nup153 and Mtor were shown to bind the genome in long stretches, termed Nup Associated Regions (NARs), which were similarly enriched for active genes (Vaquerizas et al., 2010). Such NARs further suggest that NPC components contribute to global chromatin organization, similarly to Lamins. Interestingly, *C. elegans* NPC components were recently found to associate specifically with targets of RNA Polymerase III (RNAP III), such as *tRNA* and *snoRNA* genes, where they appear to be functionally required for correct RNA processing (Ikegami and Lieb, 2013). Since expression of RNAP III targets such as *tRNA* genes has also been shown to be highly tissue specific (Dittmar et al., 2006), these findings suggest that Nups may contribute to cell fate via regulation of both RNAP II and RNAP III targets.

Intriguingly and in line with their dynamic behavior, *Drosophila* Nups have been shown to be recruited to their target genes in the nucleoplasm, away from the NE embedded NPCs, suggesting that the ability of Nups to regulate or support active chromatin can be carried out at any location in the nucleus (Capelson et al., 2010; Kalverda et al., 2010; Vaquerizas et al., 2010). Both the off pore mode of Nup-gene interactions and the binding of Nups to developmentally induced genes were recently also observed in human cells. Genome wide binding studies of human Nup98 in ESCs, neural progenitor cells and differentiated IMR90 fibroblasts revealed large tissue specific differences in Nup98 target genes and demonstrated that a subset of genes activated during ESC differentiation are recruited to the NPC (Liang et al., 2013). Together, these studies in metazoan systems support the notion that the NPC or individual Nups bind and promote activation of genes induced in a lineage specific manner, thus constituting another important NE linked complex with a role in gene expression and cell fate (**Figure 2C**). In this manner, the NPC may represent a distinct nuclear environment that promotes a permissive chromatin state at the nuclear periphery, functionally opposed to the roles of the Lamins and NETs (**Figures 2A,B**), but perhaps providing an accessible scaffold for switching between silenced and activated states during cell differentiation.

In support of the link of the nuclear pore to chromatin structure, suggested by the early EM images, several histone modifying enzyme complexes have been linked to the NPC. In addition to the reported interaction of the yeast NPC with the SAGA HAT complex (Rodriguez-Navarro et al., 2004; Cabal et al., 2006; Pascual-Garcia et al., 2008), *Drosophila* Nup98 was found to associate with histone modifying complexes such as the histone methyl transferase Trithorax (Trx), the fly homolog of Mixed Lineage Leukemia (MLL), and the Non-Specific Lethal (NSL) Complex, which carries a conserved HAT males absent on the first (MOF; Pascual-Garcia et al., 2014). MOF, as part of the fly dosage compensation complex that maintains transcriptional hyperactivity of the male X chromosome, has also been shown to associate with Nups Nup153 and Mtor (Mendjan et al., 2006). Since both Trx/MLL and NSl/MOF are critical epigenetic regulators, these interactions further implicate Nups in the epigenetic memory of transcription, suggested by yeast studies. Interestingly, the memory function of yeast Nups appears to be conserved in human cells. HeLa cells treated with interferon gamma (IFN-γ) show faster reactivation of IFN-γ inducible genes than cells never exposed to IFN-γ (Light et al., 2013), demonstrating that these genes are marked as recently transcribed. As its yeast homolog Nup100, Nup98 was found to be required for propagating this memory through cell divisions, since Nup98-depleted cells lose the enhanced transcriptional response to IFN-γ repeated exposure. In this case, chromatin structure again appears to be involved, as the deposition of histone H3 lysine K4 di-methylation at target gene promoters is gained during the memory acquisition and lost as a result of Nup98 knock down. Together, these findings highlight transcriptional and epigenetic regulation of genes by Nup binding as a likely mechanism for some of the tissue specific phenotypes of Nups and a new regulatory aspect of cell fate determination.

### Nuclear Pore Proteins Contribute to Chromatin Organization

In addition to transcribing genes, the NPC has been implicated in binding silenced genomic regions and chromatin boundary elements (**Figure 2C**). The earliest genome wide binding analysis of various Nups in yeast demonstrated that the stable yeast Nup84 (mammalian Nup107) binds to loci that are not enriched for transcriptional activity, and thus termed “neutral” chromatin (Casolari et al., 2004). Subsequently, ChIP analysis of another stable NPC component, Nup93, in human cells similarly demonstrated that the Nup93 binding targets in HeLa cells included nontranscribing regions, enriched for silent histone modifications (Brown et al., 2008). Recently, a study carried out in budding yeast revealed a direct functional involvement of the stable Nup170 (mammalian Nup155) in maintenance of silent heterochromatin (Van de Vosse et al., 2013). Nup170 was identified at repressed genomic regions such as ribosomal protein and subtelomeric genes, and was demonstrated to be required for their silencing via interactions with the chromatin remodeling remodels the structure of chromatin (RSC) complex and the silent information regulatory (SIR) complex component Sir4. It appears that the NPC can bind both active and silent genes, likely through using different Nup components, each of which has the ability to interact with different types of chromatin regulatory complexes.

Boundary elements or insulators are critical for the establishment and maintenance of the correct genome architecture in a cell type specific manner (Van Bortle and Corces, 2012). Their main property involves the ability to separate chromatin domains of varying activity states from each other, as for example, insulating euchromatin from heterochromatin. Recently, a role in delineating euchromatin and heterochromatin domains has been described for the nuclear basket Nup Tpr in mammalian culture cells (Krull et al., 2010). Depletion of Tpr resulted in the loss of the decondensed chromatin regions associated with the NPCs and allowed the spread of heterochromatin into the nuclear regions underlying the nuclear pores, as assessed by EM. Interestingly, the binding sites of the NE embedded NPCs (not the dynamic components) in fly S2 culture cells were found to be enriched for the binding sites of a well characterized insulator protein Suppressor of Hairy Wing [Su(Hw); Kalverda and Fornerod, 2010]. The ability of the NPC to function as an insulator between active and silenced regions has also been demonstrated in yeast. A genetic screen for proteins with boundary activity, using a reporter gene positioned next to a heterochromatic domain, identified several exportins and Nup2 as being able to insulate the reporter gene from silencing (Ishii et al., 2002). Additionally, several Nups such as Nup2 and Nup60 were found at the tRNA insulator of the yeast silenced HMR mating locus, although their depletion did not compromise insulating activity (Ruben et al., 2011). Binding of stable Nups was similarly reported at *tRNA* genes in *C. elegans* embryos (Ikegami and Lieb, 2013), further supporting the notion that the NPC may serve a conserved boundary function in eukaryotic genomes.

These studies lend the view of the NPC as another important scaffold for spatial genome organization (**Figure 2C**), which bears direct relevance to the establishment of cell type specific gene expression programs. Whether the metazoan NPC primarily functions as a scaffold for expression of RNAP II and RNAP III genes, for establishment of chromatin boundaries or for additional regulation of silenced genes remains to be fully deciphered. It is possible that this large protein complex can accommodate interactions with all three types of loci via different Nups. Furthermore, the stable proteins of the NPC have been shown to be remarkably long-lived. Once assembled, the NPC core essentially does not turn over during the entire life span of post-mitotic cells, such as neurons (D’Angelo et al., 2009; Toyama et al., 2013). This extreme stability makes the NPC a well suited nuclear scaffold for establishing long term genome organization and thus transcription programs.

## Interplay Between NE Components

Rather than thinking of these compartments individually, accumulating evidence portrays the NE as a machine with many components working together to affect gene expression programs and differentiation. Many of the known NE components of the nuclear lamina, the NPC and the NM are known to associate with each other, and this high level of interplay makes it difficult to separate the functions of these compartments. Current work indicates that Lamin A isoforms interact with integral and associated NM proteins, which are expressed in a tissue specific manner, thus further contributing to tissue specific genome conformations and gene expression profiles. As discussed above, Lamins have been shown to interact with several NM proteins including LBR, Emerin, Man1, Lap2a as well as barrier to autointegration factor (BAF; Ho and Lammerding, 2012). These interactions are required for many of the reported NE-genome contacts and for supporting repressive effects that the nuclear lamina can exert on gene expression. Additionally, Lamins are known to associate with the Sun and Nesprin proteins, which form the LINC complex connecting chromatin and the Lamina to the cytoskeleton. Nuclear envelope retention of some of these proteins, such as Emerin, as well as a subset of less characterized NETs has been shown to be Lamin A dependent (Sullivan et al., 1999; Malik et al., 2010).

Nuclear pore complex components such as Nup153 and Nup88 have also been shown to interact with the Lamins (Ho and Lammerding, 2012). But although they appear to contact each other closely in nuclear space, the precise molecular relationship between nuclear lamina and nuclear pores is still unclear. A recent study provided an example of this relationship in the *Drosophila* testes stem cell niche, where Lamin Dm0 was found to regulate ERK and epidermal growth factor (EGF) receptor signaling to maintain cyst stem cells and support differentiation of germ stem cells (Chen et al., 2013). This function of Lamin is carried out via Nups, such as Nup153, which results in nuclear retention of phosphorylated ERK in the cyst stem cells. Here, the nuclear lamina appears to contribute to setting up the correct composition of the NPC, which in turn regulates developmental EGF signaling to control the stem cell niche. Future studies of the interplay between nuclear lamina, the NPC, the INM proteins and the LINC complex components are sure to yield exciting new aspects of developmental regulation.

## Conclusion

Accumulating evidence has demonstrated tissue specific presence and functions of various NE components. Much of that knowledge supports the model that many of these functions are carried out via cell type specific interactions between the NE and the genome, which contribute to the correct establishment of tissue specific gene expression (**Figure 2**). Tissue specific expression of Lamin isotypes appears to be important for tethering heterochromatin to the nuclear periphery and for repositioning critical developmental genes to a silencing nuclear compartment (**Figure 2A**). This role of nuclear lamina is closely linked to and likely executed through the functions of INM proteins, which have the ability to interact with chromatin bound regulators and histone modifying complexes. Expression of NETs has been shown to be highly cell type specific and likely drives the reorganization of chromosomes and large genomic regions needed for certain paths of differentiation (**Figure 2B**). Finally, components of the NPC are functionally implicated in regulation of developmentally induced active genes and in setting up boundaries between chromatin domains (**Figure 2C**). The composition of the NPC may vary depending on the cell type, with some Nups such as Nup210 being added to drive critical differentiation steps. Developmentally regulated genes and boundaries may thus be recruited to the NPC in a tissue specific manner, or recruit Nups to their intranuclear locations. An exciting new direction stemming from these models is how developmental signaling factors that enter the nucleus during differentiation of particular lineages may cofunction with NE proteins and influence their genomic binding.

Together the presented data also illustrate the interconnected roles of nuclear compartments essential for cell fate determination, from the earliest steps of chromatin structure rearrangement to the last stages of morphological and other changes. Perhaps a more accurate view of the NE-genome interplay involves a myriad of overlapping mechanisms with increasing specificity during differentiation. The NE composition may be another “cellular code” for specifying tissue specific gene expression programs through its contacts with the underlying chromatin. Similarly to other highly complex regulatory networks, future applications of the systems biology view of this “code” may be particularly beneficial for fully understanding the role of the NE in genome function and cell fate.
